# Patterns and socio-demographic correlates of domain-specific physical activities and their associations with adiposity in the China Kadoorie Biobank study

**DOI:** 10.1186/1471-2458-14-826

**Published:** 2014-08-09

**Authors:** Huaidong Du, Liming Li, Gary Whitlock, Derrick Bennett, Yu Guo, Zheng Bian, Junshi Chen, Paul Sherliker, Ying Huang, Ningmei Zhang, Xiangyang Zheng, Zhongxiao Li, Ruying Hu, Rory Collins, Richard Peto, Zhengming Chen, on behalf of the China Kadoorie Biobank (CKB) collaborative group

**Affiliations:** Clinical Trial Service Unit and Epidemiological Studies Unit (CTSU), Nuffield Department of Population Health, University of Oxford, Oxford, UK; School of Public Health, Peking University Health Sciences Center, Beijing, China; Chinese Academy of Medical Sciences, Beijing, China; China National Center for Food Safety Risk Assessment, Beijing, China; Non-Communicable Disease Prevention and Control Department, Provincial Center for Disease Control, Guangxi Zhuang Autonomous Region, China; Non-Communicable Disease Prevention and Control Department, Provincial Center for Disease Control, Sichuan Province, China; Non-Communicable Disease Prevention and Control Department, Meilan Center for Disease Control, Hainan Province, China; Non-Communicable Disease Prevention and Control Department, Maiji Center for Disease Control, Tianshui, Gansu Province China; Administration office, Provincial Center for Disease Control, Zhejiang Province, China

**Keywords:** Physical activity, Domain, Obesity, China, Biobank

## Abstract

**Background:**

Domain-specific physical activities may have different correlates and health effects, but few large studies have examined these questions, especially their separate associations with adiposity.

**Methods:**

We analysed cross-sectional data of 466 605 adults without any prior chronic diseases, enrolled during 2004–8, from 10 diverse localities across China. Physical activity level in each of 4 domains (occupation, commuting, household, and active-recreation), calculated as metabolic equivalent (MET)-hr/day, was related to social-demographic factors and measures of adiposity (body mass index [BMI], waist circumference [WC], and bio-impedance derived percentage body fat), using multivariable linear and logistic regression models.

**Results:**

The overall mean age was 50.8 years. The mean total physical activity was 21.7 MET-hr/day, mainly from occupation (62%) and household chores (26%), but little from active-recreation (4%), with women having a much higher household activity than men. Older participants had a lower level of occupational activity but a higher level of household and active-recreational activity, particularly after retirement. There was no linear association of occupational activity with adiposity, but working women tended to have a lower adiposity (e.g. 1.0 cm WC) than non-working women. In men, there was an inverse and apparently linear association between adiposity and levels of both commuting-related and household activities, with 3 MET-hr/day associated with -0.11 and -0.13 kg/m^2^ BMI, -0.42 and -0.62 cm WC, and -0.28 and -0.33 percentage points of body fat, respectively. In women, only household activity showed a linear, but weaker, association with adiposity. A higher adiposity was observed among men and women with higher levels of active-recreational activity.

**Conclusions:**

In Chinese adults, physical activity mainly involves occupation and housework, with little from active-recreational activity. Domain-specific physical activities varied by socio-demographic factors and had different associations with adiposity.

**Electronic supplementary material:**

The online version of this article (doi:10.1186/1471-2458-14-826) contains supplementary material, which is available to authorized users.

## Background

The health benefits of physical activity have been well recognized [1]. However, studies to date have focused primarily on active-recreational activities, in spite of a large contribution to total physical activity level of occupational, household and commuting-related activities, particularly in low- and middle-income countries, such as China [2–4]. Activities from different domains may differ in their determinants [5–7] and knowledge about them could help to inform the development of effective physical activity interventions at a population level. Several studies have examined associations of physical activity from different domains with socio-demographic factors, but these were mainly in Western countries where people in general have an urban-industrial lifestyle. In China, although there has been a large shift from a labour-intensive lifestyle to a more sedentary lifestyle over the past few decades, patterns of physical activity still differ greatly from those in the Western countries, with, for example, higher participation in leisure-time and household activity among older people in China [8], contrasting that seen in most Western populations [6].

A higher level of physical activity has been deemed as beneficial for preventing weight gain and obesity. Question remains, however, about the separate associations of domain-specific physical activities with adiposity and other health-related outcomes. Several studies have attempted to address these issues but the findings were inconsistent, with some showing inverse association between commuting-related activity and adiposity in various populations including Chinese [9], South Asians [10], and Americans [11] whereas others showing no association [12], an inverse association [2, 13], or a positive association [14] between occupational activity and adiposity. Large-scale epidemiological studies comparing simultaneously the associations of physical activity from four major domains with measures of adiposity are still lacking.

To fill this evidence gap, we report the findings of a cross-sectional investigation using data from the China Kadoorie Biobank (CKB), a prospective observational study of over 500 000 adults recruited from 10 diverse areas in China [15, 16]. The aims of this report are: 1) to determine the socio-demographic correlates of physical activity from occupation, commuting, housework and active- recreation; 2) to investigate the associations of domain-specific activity with different measures of adiposity including body mass index (BMI), waist circumference (WC) and percentage body fat.

## Methods

### Study design and participants

Detailed information about the CKB study design, survey methods and participant characteristics has been reported previously [15, 16]. In brief, all permanent residents aged 35–74 years in 10 geographically defined areas (5 urban and 5 rural) of China, identified through public registry records, were eligible to take part in the study. The areas were selected so as to cover wide spectra of risk exposures and disease patterns, and for logistical convenience, but were not intended to be representative of China as a whole. The baseline survey was conducted between June 2004 and July 2008. Invitation letters and study information leaflets were delivered door-to-door by local community leaders and health workers following extensive publicity campaigns. About one in three of the eligible residents (33% rural, 27% urban) participated. To encourage participation, those few individuals aged 30–34 or 75–79 years were not turned away [16]. In total, 512 891 participants (210 222 men and nearly all Han Chinese) were enrolled in the CKB.

For the current analyses, we excluded those who had a history of heart disease (n = 15 472), stroke (n = 8884), tuberculosis (n = 7660), chronic obstructive pulmonary disease (n = 13 289), and/or cancer (n = 2577); those who answered all questions in the physical activity questionnaire with zero (n = 81); those who reported spending more than 20 hours daily on all waking activities (n = 813); and those who gave implausible or conflicting answers to occupational and commuting-related questions (e.g. who reported not working but had non-zero commuting-related physical activity) (n = 1418). After these exclusions, 466 605 participants remained in this cross-sectional analysis.

The study was approved by the ethics committees of the University of Oxford and the Chinese Center for Disease Control and Prevention (CDC). In addition, ethics approval was obtained from the institutional research board at the local CDC in each of the 10 areas. Written informed consent was obtained from all participants.

### Data collection

The baseline data collection took place in temporary assessment clinics specially set up in the local communities for this study. An interviewer-administered computerised questionnaire was used to collect information on socio-demographic factors (e.g. education, annual household income, and occupation), lifestyle factors (e.g. smoking, drinking, diet and physical activity), and medical history.

Detailed information on physical activity questions and methods used to derive domain-specific physical activity levels, in metabolic equivalents (MET)-hr/day, have been reported previously [17]. In brief, questions about physical activity were adapted from validated questionnaires used in several other studies in Western [18] and Chinese populations [19], with some additional modifications following a CKB pilot study. To quantify the amount of physical activity, metabolic equivalents (METs) from the 2011 update of a major compendium of physical activities [20] were used. Occupational activity included all physical activities performed during paid employment; commuting-related activity was the physical activity performed during the journeys between home and work, without including the transportation for other purposes; household activity included activities spent on household chores; and active-recreational activity included activities spent on sports during leisure time, but not sedentary leisure-time activities (mainly TV viewing) [17].

Body weight, standing height, WC and body fat percentage were measured by trained health workers while participants wore light clothes and no shoes. Standing height was measured to the nearest 0.1 cm using a stadiometer, and WC was measured with a soft non-stretchable tape at midway between the lowest rib and the iliac crest, also to the nearest 0.1 cm. Weight and percentage body fat were estimated using a bioelectrical impedance device (TANITA-TBF-300GS, Tanita Corp., Tokyo, Japan). BMI was calculated as the weight in kilograms divided by the square of standing height in meters.

### Statistical analyses

For each of the four domains of physical activity, participants were first divided into two groups: *performing no such activity* or *some activity*; and the proportions of participants with *no activity* by socio-demographic factors were estimated separately for men and women using logistic regression models, adjusting for age (in 5-year intervals) and study area, where appropriate. Among those who had some activity from a specific domain, multiple linear regression models were used to estimate the age- and area-adjusted mean levels of activity by socio-demographic factors.

The adjusted mean levels (and 95% CIs) of adiposity measures in 4 groups of each domain-specific physical activity (zero, low, moderate or high) were estimated using multiple linear regression models with adjustment for age, study area (10 study areas), education (no formal education, primary school, middle or high school, and college or university), annual household income (<10 000, 10 000–19 999, 20 000–34 999, and ≥35 000 yuan), sedentary leisure time (hr/day), smoking (never, ex-regular, occasional and current smokers), alcohol intake (never, ex-regular, occasional and current drinkers) and other domain-specific activities (MET-hr/day). Analyses were performed separately for men and women and, if an approximately linear association was observed, differences in adiposity per 3 MET-hr/day of domain-specific physical activity were also examined using multiple linear regression with heterogeneity assessed by χ^2^ statistics, *P* values from Cochran Q tests, and *I*^*2*^ statistics (i.e. the variation in pooled estimates accounted for by heterogeneity rather than by chance) [21]. All statistical analyses were conducted using SAS 9.2 (SAS Institute Inc., Cary, NC, USA).

## Results

Of the 466 605 participants included, the mean baseline age was 50.8 years, 40.4% were men, and 42.5% came from urban areas. Occupational activity was the largest domain, accounting for 76% and 52% of total physical activity in men and women respectively, and active-recreational activity was the smallest, accounting for less than 4% of total physical activity in both sexes. Nearly all women but only about 79% of men engaged in household work with the mean activity level in women being more than 2.5 times as high as in men (7.7 vs. 2.9 MET-hr/day). Women had higher mean percentage body fat (32% vs. 22%), higher mean BMI (23.8 vs. 23.4 kg/m^2^), but lower mean WC (78.8 vs. 81.9 cm) than men (Table 1).

**Table 1:** **Main characteristics of study participants**

Characteristics	Men (n =188 647)	Women (n = 277 958)	Total (n =188 647)
Age (years), %			
30 – 39	15.2	16.9	16.2
40 – 49	30.0	32.2	31.3
50 – 59	30.6	30.8	30.7
60 – 69	17.8	15.2	16.2
70 – 79	6.5	4.8	5.5
*Mean (SD)*	*51.5 (10.7)*	*50.3 (10.3)*	*50.8 (10.5)*
Residence, %			
Urban	42.6	43.3	43.0
Rural	57.4	56.7	57.0
Occupational physical activity (MET-hr/day), %			
0	18.1	34.7	28.0
0.1– 10	18.0	20.7	19.6
10.1 – 20	25.5	23.1	24.1
> 20	38.4	21.5	28.3
*Mean (SD)*	*17.3 (14.9)*	*10.9 (12.5)*	*13.5 (13.9)*
Commuting-related physical activity (MET-hr/day), %			
0	31.4	47.3	40.9
0.1 – 1.5	23.5	17.9	20.1
1.6 – 3.0	26.0	20.2	22.6
> 3.0	19.1	14.6	16.4
*Mean (SD)*	*1.9 (2.4)*	*1.4 (2.2)*	*1.6 (2.3)*
Household physical activity (MET-hr/day)			
0	21.0	0.7	8.9
0.1 – 4.0	54.5	15.8	31.4
4.1 – 8.0	17.4	31.8	26.0
> 8.0	7.0	51.8	33.7
*Mean (SD)*	*2.9 (2.8)*	*7.7 (3.9)*	*5.7 (4.2)*
Active-recreational physical activity (MET-hr/day), %			
0	79.0	80.4	79.8
0.1 – 2.0	6.9	5.8	6.2
2.0 – 4.0	6.8	6.5	6.7
> 4.0	7.2	7.3	7.3
*Mean (SD)*	*0.8 (2.2)*	*0.8 (2.2)*	*0.8 (2.2)*
Total physical activity (MET-hr/day)	*22.8 (15.1)*	*20.9 (12.8)*	*21.7 (13.8)*
Sedentary leisure time (hr/day)	*3.1 (1.5)*	*2.9 (1.5)*	*3.0 (1.5)*
Body mass index (kg/m^2^)^1^	*23.4 (3.2)*	*23.8 (3.4)*	*23.6 (3.3)*
Waist circumference (cm)	*81.9 (9.7)*	*78.8 (9.4)*	*80.1 (9.6)*
Percentage body fat (%)^2^	*22.0 (6.2)*	*32.0 (7.1)*	*28.0 (8.3)*

SD: standard deviation.

^1^2 participants with missing values.

^2^214 participants with missing values.

In both sexes, age was strongly and inversely associated with proportion of having and the mean levels of occupational activity. Due to retirement, the proportion of non-working people increased dramatically at 60–69 years for men (from 12.5% at 50–59 years to 49.3%) and 50–59 years for women (from 13.4% at 40–49 years to 45.6%). At the same time, mean levels of occupational activity, among those still working, greatly decreased (Table 2). An opposite trend was observed for active-recreational activity; those aged 70–79 years were ~3.6 times more likely to perform regular physical exercise than those aged 30–39 years. Their mean level of exercise was also higher than those younger than 60 years (for men) or 50 years (for women).

**Table 2:** **Levels of domain-specific physical activity by age and area** ^**1**^

Characteristics	n	Physical activity							
		Occupational		Commuting-related		Household		Active-recreational	
		% no activity	Mean MET-hr/day (SD) ^2^	% no activity ^3^	Mean MET-hr/day (SD) ^2^	% no activity	Mean MET-hr/day (SD) ^2^	% no activity	Mean MET-hr/day (SD) ^2^
**Men**									
Age, years									
30 – 39	28 694	1.7	23.6 (12.8)	17.8	2.5 (2.2)	24.0	2.9 (2.6)	88.4	3.1 (3.2)
40 – 49	56 513	2.9	23.1 (12.8)	16.7	2.6 (2.2)	22.8	3.2 (2.6)	87.5	3.2 (3.2)
50 – 59	57 721	12.5	20.9 (12.8)	15.2	2.9 (2.1)	20.9	3.7 (2.6)	79.7	3.9 (3.2)
60 – 69	33 512	49.3	15.2 (12.9)	15.6	2.8 (2.2)	16.9	4.3 (2.6)	62.7	4.5 (3.2)
70 – 79	12 207	68.1	10.3 (12.8)	16.4	2.6 (2.2)	17.5	4.3 (2.6)	58.1	4.4 (3.2)
Region									
Rural	108 347	4.8	21.5 (13.6)	19.4	2.9 (2.5)	23.7	3.8 (2.6)	91.9	2.7 (3.2)
Urban	80 300	36.1	20.4 (13.7)	10.5	2.4 (2.5)	17.4	3.4 (2.6)	61.4	4.3 (3.2)
**Women**									
Age, years									
30 – 39	47 033	9.0	18.4 (10.3)	19.4	2.6 (1.9)	1.1	6.8 (3.6)	90.5	3.0 (3.1)
40 – 49	89 625	13.4	18.4 (10.3)	19.1	2.8 (1.9)	0.7	7.1 (3.6)	87.2	3.6 (3.1)
50 – 59	85 692	45.6	14.8 (10.3)	18.9	2.8 (1.9)	0.4	8.7 (3.6)	76.6	4.5 (3.1)
60 – 69	42 206	70.1	11.7 (10.3)	21.2	2.7 (1.9)	0.6	8.4 (3.6)	66.2	4.3 (3.1)
70 – 79	13 402	85.8	8.0 (10.2)	21.3	2.6 (1.9)	1.7	7.6 (3.6)	68.4	4.0 (3.1)
Region									
Rural	157 557	17.1	15.8 (11.7)	21.7	2.9 (2.4)	0.5	8.4 (3.7)	93.9	2.8 (3.1)
Urban	120 401	57.7	18.8 (11.8)	13.8	2.5 (2.4)	0.9	7.0 (3.7)	62.7	4.4 (3.1)

^1^Values were adjusted for age and study area (where appropriate). All P for heterogeneity across subgroups < 0.0001 for all.

^2^Least square means (standard deviations) among those participants who have that domain-specific physical activity.

^3^The proportion was calculated among working people only.

Compared with rural participants, urban participants were more likely to participate in all three non-occupational activities and had a higher mean level of active-recreational activity, but lower mean levels of commuting-related and household activity. On the other hand, mean level of occupational activity was higher in rural men and urban women as compared to their corresponding counterparts. Rural farmers do not have to retire at certain ages, therefore there was a much smaller proportion of non-working people in rural than in urban areas (Table 2). Across the 10 study areas, overall mean level of occupational activity was the highest in Zhejiang (a rural costal area) in both men and women, and the lowest in men from Haikou (an urban tropical area) and women from Henan (a rural inland area). Commuting-related activity was much higher in Gansu, a rural inland area in the west of China, than in other areas. With regard to mean level of household activity, the highest was found in women from Gansu and the lowest was in men from Henan (Additional file 1: Figure S1).

Factory workers had the highest mean level of occupational activity (25.9 MET-hr/day in men and 24.1 in women), followed by sales & self-employed (21.6 in men and 21.7 in women) (Table 3). Although mean level of commuting-related activity did not vary greatly across different occupations (2.1-2.9 MET-hr/day), proportion of non-commuting sales & self-employed was 9 times (in men) or 21 times (in women) as high as that of non-commuting farmers, and 3 times (in both men and women) as high as non-commuting factory workers. The mean levels of household and active-recreational activity were the highest in those not in paid occupation, including those retired and unemployed individuals and home makers. They also had a higher tendency to perform active-recreational activity, i.e. lower proportion of no such activity. For both men and women, sales & self-employed group had the lowest rate of household activity participating. In both sexes, education was associated inversely with mean levels of occupational, commuting-related and household activity but positively with proportion engaged in active-recreational activity. A lower proportion of non-working people was observed among those with a college or university education, particularly in women. A higher level of education was associated with a lower mean level of household activity, a higher proportion of men but lower proportion of women participating in household tasks. People with college or university education were 4 times more likely to perform regular physical exercise than those with no formal education. Household income was also positively, though to a lesser extent compared with education, associated with proportion of participating in active-recreational activity. However, no clear linear association of either education or household income with mean level active-recreational activity was observed. *P* values for heterogeneity for all of these associations were <0.0001 (Table 3).

**Figure 1:**
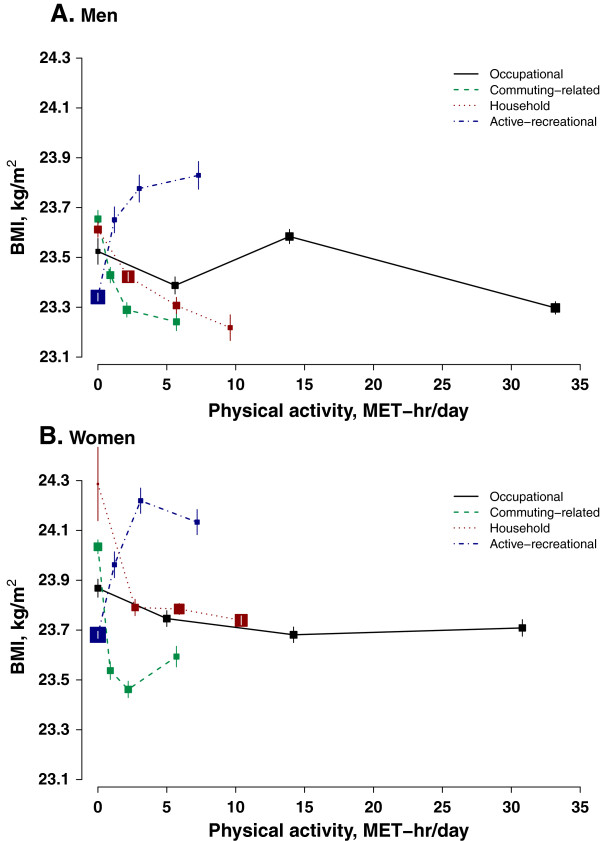
**Body mass index in relation to physical activity from different domains.** Analyses were adjusted for age (in 5-year intervals), study area, education, income, smoking, sedentary leisure time, and alcohol intake. Different domain-specific physical activities were mutually adjusted for each other. 2 participants with missing values on BMI were excluded from analyses. Sizes of boxes were inversely proportional to the standard errors of the point estimates. Panel **A** for men and panel **B** for women.

**Figure 2:**
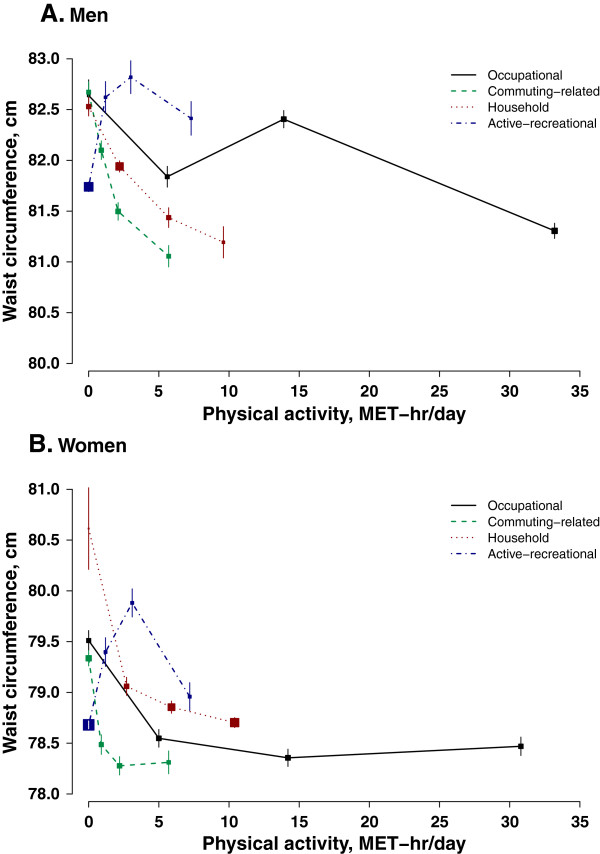
**Waist circumference in relation to physical activity from different domains.** Conventions as in Figure 1.

**Figure 3:**
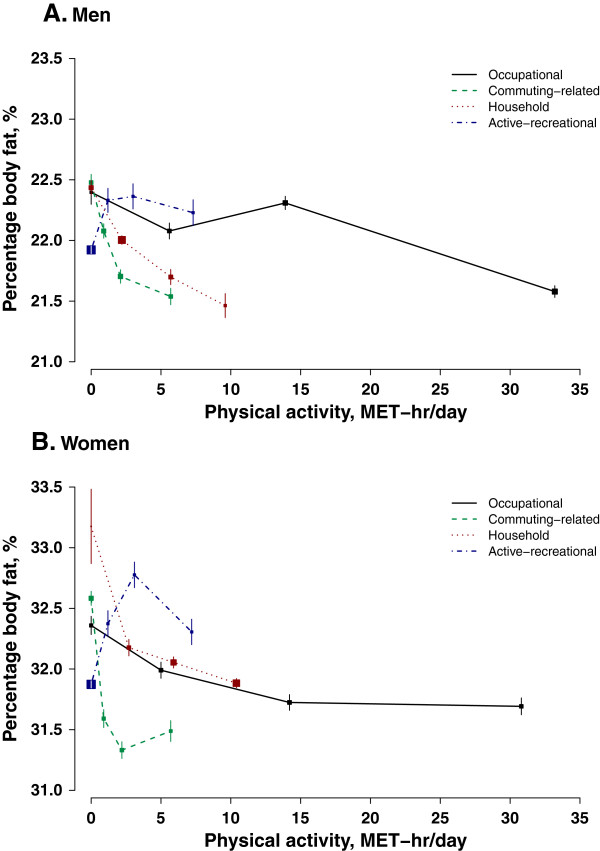
**Percentage body fat in relation to physical activity from different domains.** Conventions as in Figure 1. 214 participants with missing values on percentage of body fat were excluded from analyses.

Occupational activity was not linearly associated with any of the adiposity measures (Figure 1, Figure 2 and Figure 3). However, men with the highest level of occupational activity (i.e. > 20 MET-hr/day) had the lowest level of adiposity and women with the highest level of occupational activity also had the lowest percentage body fat. Working women were in general leaner than non-working women, in terms of all three adiposity measures, especially WC which was 1.0 cm smaller in working than in non-working women.

**Table 3:** **Levels of domain-specific physical activity by socioeconomic factors** ^**1**^

Characteristics	n	Physical activity							
		Occupational		Commuting-related		Household		Active-recreational	
		% no activity	Mean MET-hr/day (SD) ^2^	% no activity ^3^	Mean MET-hr/day (SD) ^2^	% no activity	Mean MET-hr/day (SD) ^2^	% no activity	Mean MET-hr/day (SD) ^2^
**Men**									
**Occupation**									
Farmer	84 124	1.6	19.6 (16.4)	5.7	2.9 (2.9)	20.7	3.6 (3.3)	90.3	3.3 (4.5)
Factory worker	38 389	0	25.9 (16.1)	16.9	2.6 (2.8)	19.0	3.3 (3.1)	79.3	3.2 (3.7)
Sales & others^4^	19 450	0	21.6 (13.6)	52.4	2.6 (2.3)	28.2	3.2 (2.7)	76.0	3.4 (3.3)
Professionals	13 839	0	16.4 (13.7)	27.2	2.1 (2.4)	21.5	3.1 (2.7)	61.5	3.4 (3.4)
Retired & others^5^	32 845	100	-	-	-	19.6	4.4 (3.1)	58.7	4.6 (3.8)
**Highest education**									
No formal education	16 387	11.4	22.5 (14.2)	11.8	3.1 (2.4)	22.2	3.8 (2.8)	91.1	3.8 (3.3)
Primary school	62 101	15.7	22.2 (14.0)	14.4	2.9 (2.4)	20.9	3.8 (2.8)	85.7	3.8 (3.4)
Middle or high school	95 869	21.6	21.0 (13.7)	17.9	2.6 (2.3)	21.0	3.5 (2.7)	75.5	4.0 (3.2)
College or university	14 290	13.0	14.8 (13.9)	19.0	2.2 (2.4)	19.8	3.2 (2.8)	59.0	3.9 (3.3)
**Annual household income (yuan)**									
<10 000	48 718	20.0	21.8 (15.3)	13.2	3.0 (2.6)	18.1	4.0 (3.0)	86.7	3.8 (3.4)
10 000–19 999	53 248	21.3	22.3 (13.0)	15.0	2.8 (2.2)	21.6	3.7 (2.6)	80.4	4.0 (3.2)
20 000–34 999	48 151	16.3	21.4 (13.6)	15.6	2.6 (2.3)	21.0	3.5 (2.7)	77.1	3.9 (3.2)
≥35 000	38 530	13.6	18.6 (14.1)	22.5	2.3 (2.4)	23.9	3.2 (2.8)	69.5	3.9 (3.3)^6^
**Women**									
**Occupation**									
Farmer	116 776	2.2	13.8 (12.5)	3.6	2.8 (2.5)	0.2	8.0 (4.4)	92.3	3.4 (4.6)
Factory worker	31 190	0	24.1 (12.2)	25.1	2.7 (2.4)	1.3	5.4 (3.9)	83.4	3.1 (3.4)
Sales & others^4^	24 826	0	21.7 (11.6)	74.3	2.8 (2.2)	2.3	5.9 (3.7)	83.4	3.2 (3.2)
Professionals	11 304	0	15.0 (11.1)	41.6	2.5 (2.2)	1.6	5.5 (3.6)	67.8	3.2 (3.3)
Retired & others^5^	93 862	100	-	-	-	0.5	9.1 (4.1)	65.4	4.5 (3.4)
**Highest education**									
No formal education	70 147	27.8	16.5 (12.5)	18.2	3.0 (2.4)	0.6	8.1 (4.4)	90.7	3.7 (3.5)
Primary school	87 196	35.2	17.4 (10.7)	19.3	2.8 (2.0)	0.6	7.9 (3.8)	84.7	4.0 (3.3)
Middle or high school	108 625	40.9	17.1 (11.7)	20.2	2.6 (2.2)	0.7	7.6 (4.1)	72.4	4.3 (3.3)
College or university	11 990	14.2	11.4 (11.4)	18.3	2.3 (2.2)	1.1	6.4 (3.9)	61.8	3.9 (3.2)^6^
**Annual household income (yuan)**									
<10 000	82 776	32.5	16.5 (12.3)	19.8	2.8 (2.4)	0.5	7.7 (4.2)	86.6	4.0 (3.3)
10 000–19 999	81 908	37.2	17.1 (10.4)	17.7	2.8 (1.9)	0.6	7.9 (3.7)	81.8	4.1 (3.1)
20 000–34 999	67 527	33.6	17.3 (11.3)	17.0	2.7 (2.1)	0.7	7.7 (3.8)	78.1	4.1 (3.1)
≥35 000	45 747	35.7	15.7 (11.3)	24.4	2.5 (2.1)	1.1	7.7 (3.9)^6^	70.3	4.1 (3.2)^*6*^

^1^Values were adjusted for age and study area, *P* for heterogeneity < 0.0001 for all domain-specific physical activities.

^2^Least square means (standard deviations) among those participants who have that domain-specific physical activity.

^3^The proportion was calculated among working people only.

^4^Including sales, self-employed and people in other un-specified occupations.

^5^Including those retired, un-employed and home makers.

^6^ *P* for trend across subgroups > 0.05, all other *P* < 0.0001.

In men, both commuting-related activity and household activity were inversely associated with adiposity regardless of how it is measured (Figure 1A, Figure 2A and Figure 3A). Men with high level of commuting (mean 5.7 MET-hr/day) had 0.4 kg/m^2^ lower BMI, 1.6 cm smaller WC, and 1.0% lower percentage body fat than those not commuting. Similar difference in adiposity was also seen in men between high (9.6 MET-hr/day) and no household activity: 0.4 kg/m^2^, 1.3 cm, and 1.0%, respectively. As shown in Additional file 2: Figure S2, per 3 MET-hr/day higher commuting-related activity was associated with 0.13 (95% CI: 0.11, 0.15) kg/m^2^ lower BMI, 0.62 (0.56, 0.68) cm smaller WC, and 0.16 (0.12, 0.20) percentage point less body fat. Household activity had a similar association with BMI and percentage body fat (3 MET-hr/day was associated with -0.11 (-0.10, -0.13) kg/m^2^ and -0.18 (-0.15, -0.21) percentage point), but its association with WC was much weaker compared to that of commuting-related activity (3 MET-hr/day was related to -0.42 (-0.38, -0.47) cm).

Female commuters overall had a much lower degree of adiposity than those who did not commute (0.5 kg/m^2^ BMI, 1.0 cm WC, and 1.1% body fat). However, there was a non- linear association between commuting-related activity and adiposity and women who had a moderate amount of commuting-related activity (1.6-3.0 MET-hr/day) tended to have the lowest level of adiposity (Figure 1B, Figure 2B and Figure 3B). In women, the inverse association between household activity and adiposity, though statistically significant, was much weaker than that in men. Restricting the analyses only to women who had some household activity (only 0.7% women were excluded), each 3 MET-hr/day higher household activity was associated with 0.02 (0.01, 0.03) kg/m^2^ lower BMI, 0.09 (0.06, 0.12) cm smaller WC, and 0.07 (0.05, 0.10) percentage point less body fat (Additional file 3: Figure S3).

In both men and women, those who reported performing some regular exercise during their leisure time tended to have a higher level of adiposity than those who did not (0.4 kg/m^2^ BMI, 0.9 cm WC and 0.4% body fat in men and 0.4 kg/m^2^ BMI, 0.7 cm WC and 0.6% body fat in women) (Figure 1, Figure 2 and Figure 3).

## Discussion

This is the first well-powered and detailed study of domain-specific physical activity in relation to socio-demographic factors and adiposity among Chinese men and women from both urban and rural areas. With a much larger and more diverse population, our study confirms the findings reported in most previous studies based on people from China and other low- and middle-income countries [8, 22–24] in the following aspects: occupational and household physical activity are the two largest domains and women on average had a much greater level of household activity than men; older participants had a lower level of occupational activity but a higher level of household and active-recreational activity, particularly after retirement, due perhaps mainly to more available time for such activities. This study also found that the proportion of people performing active-recreational activity (22% of our participants) among Chinese adults is much lower than that typically seen in Western populations [25, 26], which may be attributable partly to less available time and partly to a lack of awareness about the importance of such activity, especially in rural areas [8, 22].

Although our study population is not intended to be representative of China as a whole or of any particular province [16], the associations observed, e.g. household activity in relation to adiposity, may still be generalisable to other settings or populations at large [27, 28]. The true association might even be stronger than that observed in this study since the 10 study areas probably do not include China’s full regional extremes.

An unexpected finding in our study was the lack of apparent association of occupational activity, the largest domain of total physical activity, with adiposity. Previous studies have provided mixed findings on this. For example, in two longitudinal studies, one involving ~3500 adults from the US National Health and Nutrition Examination Survey (NHANES) [13] and one on ~9400 adults in the China Health and Nutrition Survey (CHNS) [2], there was an inverse association between occupational activity and risk of obesity. But in a cross-sectional study of 12 044 people in the Spanish National Health Survey, neither BMI nor risk of obesity was significantly associated with level of work-related physical activity [12]. In another cross-sectional study of 1745 Swedish adults, there was a positive association of occupational physical activity with adiposity in women, but not in men [14]. Conversely, in a study among 1978 Vietnamese, an inverse association was observed only in men but not in women [29]. Disparities between these findings might be due to differences in study populations and methodologies used to assess occupational activity. Moreover, residual confounding by other factors, such as work-related stress and dietary habits, might also be an explanation for the lack of association between occupational activity and adiposity in our and other studies.

Another counterintuitive finding was that participants who engaged in regular active-recreational activities had higher, instead of lower, adiposity levels than those who did not. Reporting bias might be an explanation for this as it is known that people with a higher BMI tend to over-report the amount of exercise they performed to a greater extent than those with an ‘optimal’ body weight [30]. On the other hand, overweight and obese people may have started exercise more in order to control their body weight. However, this ‘reverse causality’ cannot be investigated reliably in the present cross-sectional analysis.

As in the present study, several previous studies have also reported an inverse association between household activity and adiposity in different populations [23, 31]. In a large national cohort study of over 70 000 Thai adults, the risk of being obese was 33% lower in participants who reported doing daily household activities than those who never or rarely did so [31]. In another study of 686 Malay participants, those with the lowest amount of household activity had double the risk of obesity compared to those with the highest amount [23]. In our study, women on average spent about 2.7 hours on household-related tasks, compared with only about 1 hour for men. If men could on average spend one extra hour daily on similar activities (2.8 MET-hr/day), i.e. switching from sedentary leisure time, then their adiposity indices would improve by 0.1 kg/m^2^, 0.37 cm and 0.17 percentage point lower respectively, independent of the effects of reduced sedentary leisure time [17].

As in other similar large-scale epidemiological studies, the present study used a questionnaire to assess levels of physical activity from all domains. Compared to more objective measures of physical activity (such as accelerometers) [32], questionnaire-assessed activities are more prone to measurement error due to recall bias (particularly among the elderly), social desirability bias, and difficulties associated with capturing low-intensity activities. In addition, misclassification might have occurred because the same MET value, from a compendium of MET values which mainly came from Western studies [33], being assigned to each type of activity regardless of between-person differences in intensity of performance. Physical activity questionnaires, however, remain as the most practical, feasible and cost-effective methods for quantifying habitual physical activity from different domains in large-scale epidemiological studies involving free-living participants [34]. Although no separate validation study has been conducted on our questionnaire, the observed associations between physical activity and socio-demographic factors in our study provide support for the validity of our physical activity questionnaire.

## Conclusions

In Chinese adults, physical activity was derived mainly from occupation and housework, with active-recreational activity accounting for only a small proportion of total physical activity. Domain-specific physical activity varied by socio-demographic factors (such as sex, age, and socioeconomic status), and also had different associations with adiposity. Our findings may suggest that as well as total physical activity it is important to consider physical activity from different domains when assessing their health-related effects and recommending strategies for public health interventions. In particular, household and commuting-related activity (such as cycling) should be more widely publicized as effective ways for weight control. The continuation of the follow up for a wide range of health-related outcomes in the CKB participants will soon allow for the prospective assessment of the effects of total and domain-specific activities on risks of chronic diseases. Findings from prospective studies will further inform the development of public health policies on physical activity in China and other low- and middle-income countries where chronic disease burden is increasing.

## Electronic supplementary material

Additional file 1: Figure S1: Levels of physical activity from different domains across 10 study areas. U: Urban areas; R: Rural areas. Levels of physical activity in each area was adjusted for age (in 5-year intervals). (PDF 20 KB)

Additional file 2: Figure S2: Commuting-related and household activity with BMI, waist circumference and percentage body fat in men. Associations were expressed as the regression coefficients (ß) for 3 MET-hr/day; Overall associations were estimated as inverse-variance-weighted averages. Analyses were adjusted for age, study area, education, income, sedentary leisure time, smoking, alcohol intake and other domain-specific activities. Only participants with some physical activity were included in analyses. (PDF 7 KB)

Additional file 3: Figure S3: Household activity with BMI, waist circumference and percentage body fat in 276 078 women. Conventions as in Additional file 2: Figure S2. (PDF 6 KB)

## Abbreviations

BMI: Body mass index
CKB: China Kadoorie Biobank
MET: Metabolic equivalent
WC: Waist circumference.
