# Supplementary material for: Patterns and socio-demographic correlates of domain-specific physical activities and their associations with adiposity in the China Kadoorie Biobank study
Source: BMC Public Health. 2014 Aug 9;14:826. doi: 10.1186/1471-2458-14-826 (PMC4138397; doi:10.1186/1471-2458-14-826)

Additional file 1: Figure S1. Levels of physical activity from different domains across 10 study areas

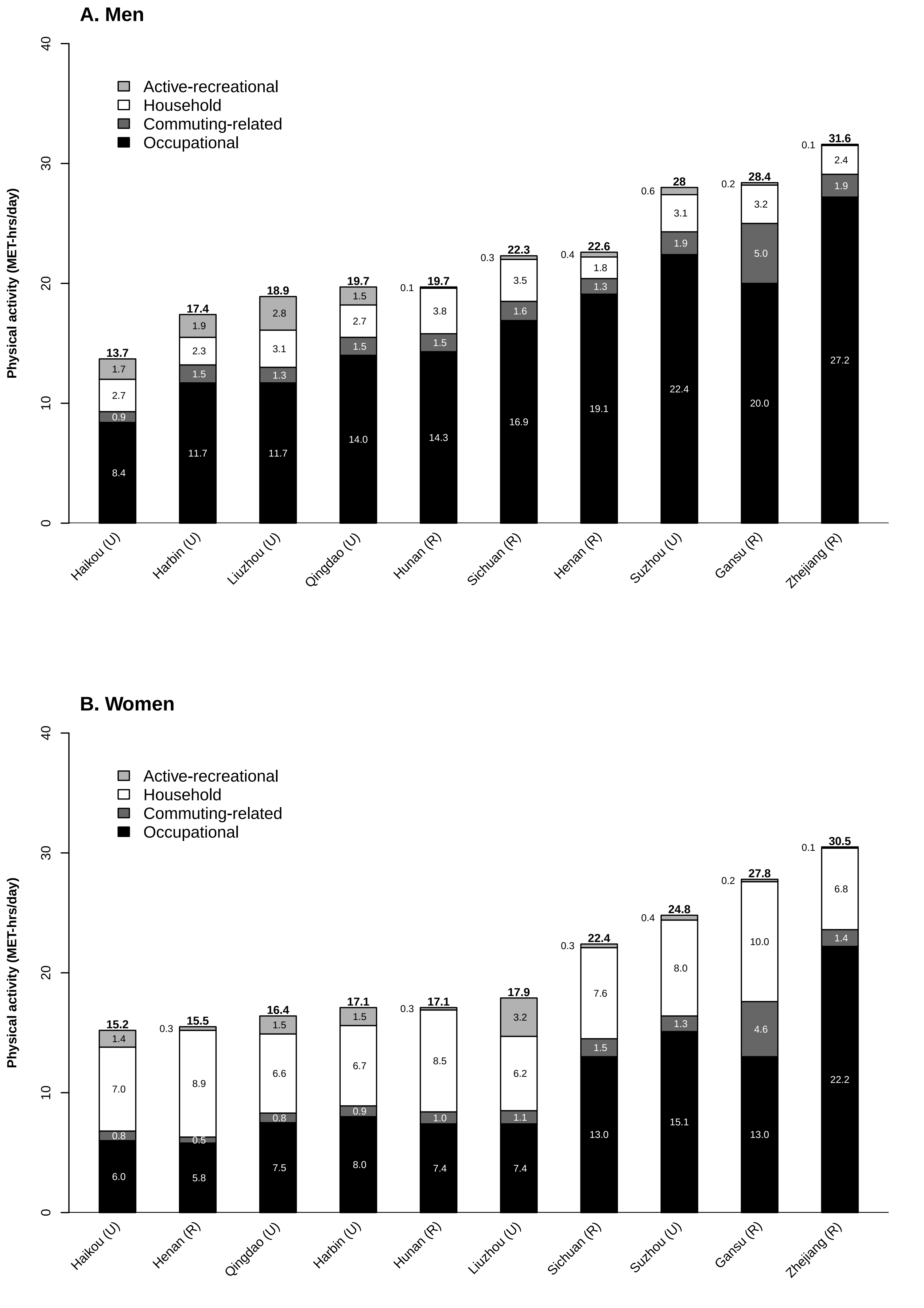

Supplement: Supplementary file 1 — Additional file 1: Figure S1: Levels of physical activity from different domains across 10 study areas. U: Urban areas; R: Rural areas. Levels of physical activity in each area was adjusted for age (in 5-year intervals). (PDF 20 KB) [file 12889_2014_6953_MOESM1_ESM.pdf]
