# Supplementary material for: Patterns and socio-demographic correlates of domain-specific physical activities and their associations with adiposity in the China Kadoorie Biobank study
Source: BMC Public Health. 2014 Aug 9;14:826. doi: 10.1186/1471-2458-14-826 (PMC4138397; doi:10.1186/1471-2458-14-826)
Supplement: Supplementary file 2 — Additional file 2: Figure S2: Commuting-related and household activity with BMI, waist circumference and percentage body fat in men. Associations were expressed as the regression coefficients (ß) for 3 MET-hr/day; Overall associations were estimated as inverse-variance-weighted averages. Analyses were adjusted for age, study area, education, income, sedentary leisure time, smoking, alcohol intake and other domain-specific activities. Only participants with some physical activity were included in analyses. (PDF 7 KB) [file 12889_2014_6953_MOESM2_ESM.pdf]

Additional file 2: Figure S2. Associations of commuting−related and household activity with BMI, waist circumference and percentage body fat in men with no history of major disease

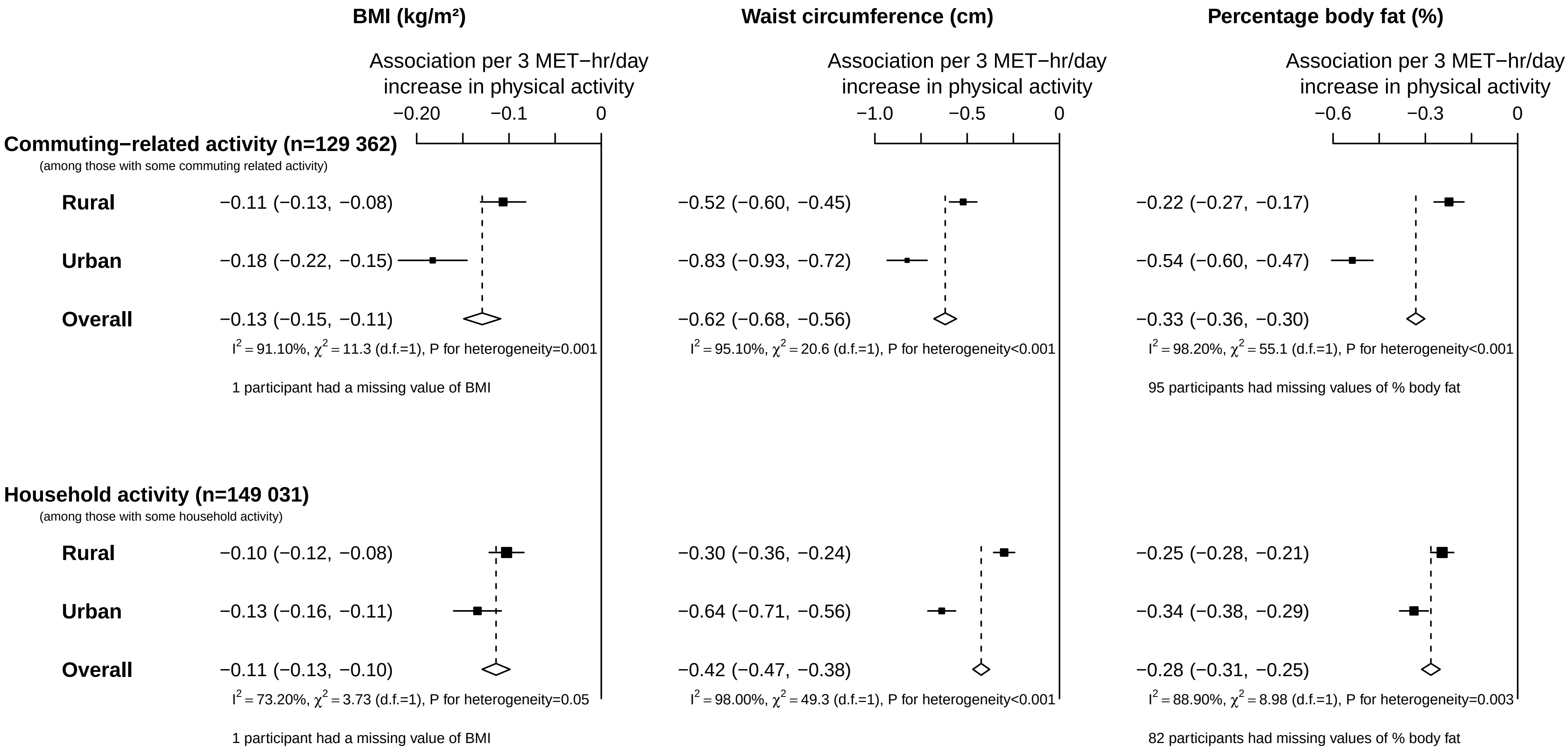

Associations were expressed as the regression coefficients (ß) for 3 MET−hr/day

Overall associations were estimated as inverse−variance−weighted averages
