# Supplementary material for: Patterns and socio-demographic correlates of domain-specific physical activities and their associations with adiposity in the China Kadoorie Biobank study
Source: BMC Public Health. 2014 Aug 9;14:826. doi: 10.1186/1471-2458-14-826 (PMC4138397; doi:10.1186/1471-2458-14-826)
Supplement: Supplementary file 3 — Additional file 3: Figure S3: Household activity with BMI, waist circumference and percentage body fat in 276 078 women. Conventions as in Additional file 2: Figure S2. (PDF 6 KB) [file 12889_2014_6953_MOESM3_ESM.pdf]

Additional file 3: Figure S3. Association of household activity with BMI, waist circumference and percentage body fat in 276 078 women with no history of major disease

A. BMI (kg/m²)

Region	N	Difference (95% CI)
**Rural**	156 774	−0.03 (−0.04, −0.01)
**Urban**	119 303	−0.004 (−0.02, 0.01)
**Overall**	276 077	−0.02 (−0.03, −0.01)

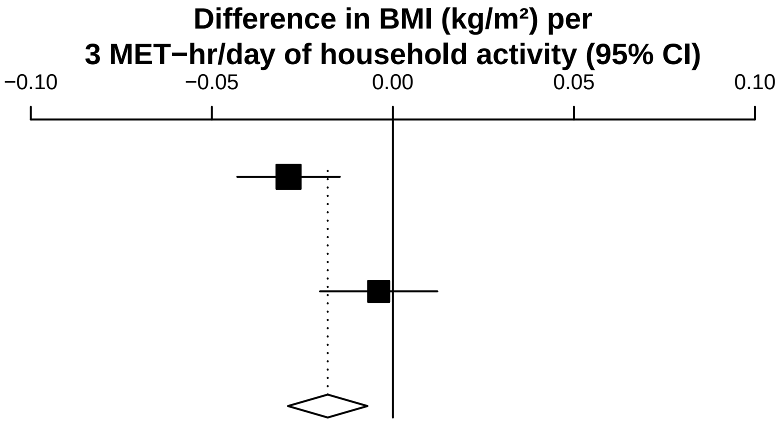

$I^2 = 80.5\%$, $\chi^2 = 5.14$ (d.f.=1), P for heterogeneity=0.023

1 participant had a missing value of BMI

B. Waist circumference (cm)

Region	N	Difference (95% CI)
**Rural**	156 775	−0.13 (−0.17, −0.09)
**Urban**	119 303	−0.04 (−0.09, 0.00)
**Overall**	276 078	−0.09 (−0.12, −0.06)

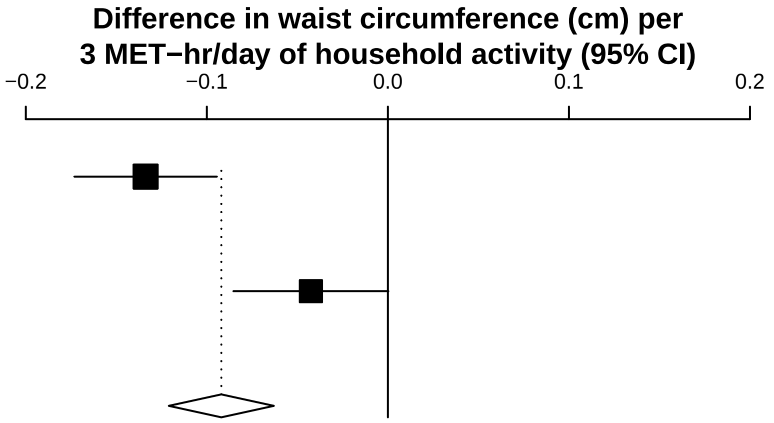

$I^2 = 89.4\%$, $\chi^2 = 9.47$ (d.f.=1), P for heterogeneity=0.002

C. Percentage body fat

Region	N	Difference (95% CI)
**Rural**	156 677	−0.07 (−0.10, −0.04)
**Urban**	119 300	−0.08 (−0.11, −0.04)
**Overall**	275 977	−0.07 (−0.10, −0.05)

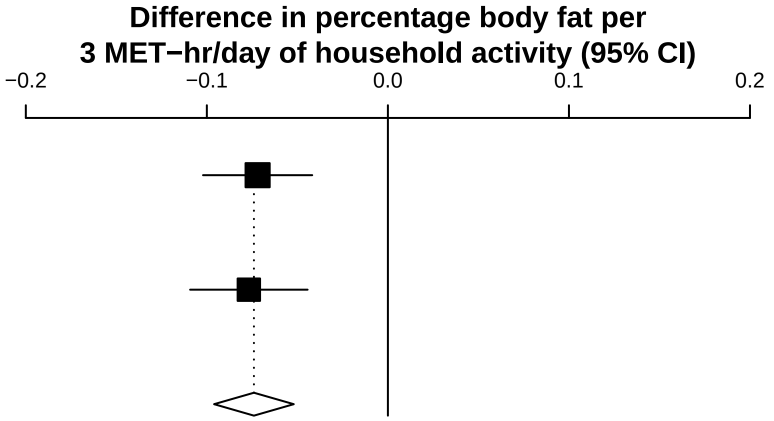

$I^2 = 0.0\%$, $\chi^2 = 0.05$ (d.f.=1), P for heterogeneity=0.83

102 participants had missing values of percentage body fat

Only those women who have some household activity were included in the analysis

Overall associations were estimated as inverse−variance−weighted averages
